# Decoding of Methylated Histone H3 Tail by the Pygo-BCL9 Wnt Signaling Complex

**DOI:** 10.1016/j.molcel.2008.03.011

**Published:** 2008-05-23

**Authors:** Marc Fiedler, María José Sánchez-Barrena, Maxim Nekrasov, Juliusz Mieszczanek, Vladimir Rybin, Jürg Müller, Phil Evans, Mariann Bienz

**Affiliations:** 1 MRC Laboratory of Molecular Biology, Hills Road, Cambridge CB2 0QH, UK; 2 EMBL, Meyerhofstrasse 1, 69117 Heidelberg, Germany

**Keywords:** DNA, SIGNALING

## Abstract

Pygo and BCL9/Legless transduce the Wnt signal by promoting the transcriptional activity of β-catenin/Armadillo in normal and malignant cells. We show that human and *Drosophila* Pygo PHD fingers associate with their cognate HD1 domains from BCL9/Legless to bind specifically to the histone H3 tail methylated at lysine 4 (H3K4me). The crystal structures of ternary complexes between PHD, HD1, and two different H3K4me peptides reveal a unique mode of histone tail recognition: efficient histone binding requires HD1 association, and the PHD-HD1 complex binds preferentially to H3K4me2 while displaying insensitivity to methylation of H3R2. Therefore, this is a prime example of histone tail binding by a PHD finger (of Pygo) being modulated by a cofactor (BCL9/Legless). Rescue experiments in *Drosophila* indicate that Wnt signaling outputs depend on histone decoding. The specificity of this process provided by the Pygo-BCL9/Legless complex suggests that this complex facilitates an early step in the transition from gene silence to Wnt-induced transcription.

## Introduction

The canonical Wnt signaling pathway controls numerous transcriptional switches during the normal development of animals, and also the homeostatic self-renewal of adult tissues (Clevers, 2006). Inappropriate activation of this pathway often leads to cancer, especially in the intestinal epithelium (Bienz and Clevers, 2000; Polakis, 2000). The Wnt-induced transcriptional switches during normal and malignant development are operated by β-catenin, or *Drosophila* Armadillo: this key effector of the pathway is activated and stabilized in response to Wnt signaling, and thus binds to TCF/LEF DNA-binding proteins to coactivate the transcription of Wnt target genes (Arce et al., 2006). This process depends on the recruitment of a wide range of different transcriptional coregulators, including chromatin modifier and remodeling complexes, to the C terminus of β-catenin associated with TCF at Wnt target genes (Willert and Jones, 2006). Notably, these include a SET1-type methyltransferase complex that promotes the trimethylation of lysine 4 in the tail of histone H3 (H3K4) (Sierra et al., 2006).

Pygopus (Pygo) and Legless (Lgs) were discovered in *Drosophila* as new Wnt signaling components that are essential for Armadillo-mediated transcription during normal development (Belenkaya et al., 2002; Kramps et al., 2002; Parker et al., 2002; Thompson et al., 2002). Similarly, their mammalian orthologs (Pygo1 and Pygo2, and BCL9 and BCL9-2/B9L, respectively) contribute to efficient β-catenin-mediated transcription in Wnt-stimulated mammalian cells, and in colorectal cancer cell lines with elevated Wnt pathway activity (Adachi et al., 2004; Brembeck et al., 2004; Thompson et al., 2002). Likewise, during vertebrate development, Pygo proteins modulate Wnt/β-catenin responses in multiple tissues (Lake and Kao, 2003; Li et al., 2007a; Schwab et al., 2007; Song et al., 2007).

Molecularly, BCL9/Lgs proteins function as adaptors between Pygo and Armadillo/β-catenin, by binding through their homology domain 1 (HD1) to the PHD finger in the C terminus of Pygo, and through their homology domain 2 (HD2) to the Armadillo repeat domain of Armadillo/β-catenin (Kramps et al., 2002; Städeli and Basler, 2005). These interactions are essential for Wnt responses during normal *Drosophila* development (Hoffmans and Basler, 2004; Townsley et al., 2004b). It was proposed that the Pygo-BCL9 complex recruits an unknown transcriptional cofactor to Armadillo/β-catenin to support Wnt-induced transcription (Hoffmans et al., 2005; Kramps et al., 2002; Thompson, 2004). Alternatively, this complex may facilitate efficient targeting of Armadillo/β-catenin to TCF target genes with which Pygo is associated even in the absence of Wnt signaling (de la Roche and Bienz, 2007; Townsley et al., 2004a), possibly reflecting an ability of its PHD finger to associate with chromatin (Bienz, 2006). Indeed, it was discovered recently that several PHD fingers can bind with high specificity and selectivity to trimethylated lysine 4 of histone H3 (H3K4me3), namely BPTF (Li et al., 2006; Wysocka et al., 2006), ING2/Yng1 (Pena et al., 2006; Shi et al., 2006, 2007; Taverna et al., 2006) and RAG2 (Ramon-Maiques et al., 2007). By contrast, the PHD finger of BHC80 binds to unmethylated histone H3 tail (H3K4me0) (Lan et al., 2007). Different types of PHD fingers thus act to read the histone code (Jenuwein and Allis, 2001; Kouzarides, 2007), and in particular the methylation status of specific histone tails (Corsini and Sattler, 2007; Taverna et al., 2007).

Here, we report the crystal structure of the human PHD-HD1 complex. We show that complex formation is critical for high-affinity binding of human and *Drosophila* Pygo PHD fingers to H3K4me, and that the binding of Pygo to methylated histone H3 tail is physiologically relevant during *Drosophila* development. The structure of two ternary complexes between PHD, HD1, and differently methylated histone H3 peptides reveals that the Pygo1 PHD finger has two distinct surfaces that are engaged simultaneously in binding to HD1 and H3K4me. Our work pinpoints three unique features of this finger: preference for H3K4me2, insensitivity to methylation of arginine 2 (H3R2), and assistance by HD1 in its recognition of the methylated histone H3 tail.

## Results

**Figure 1:**
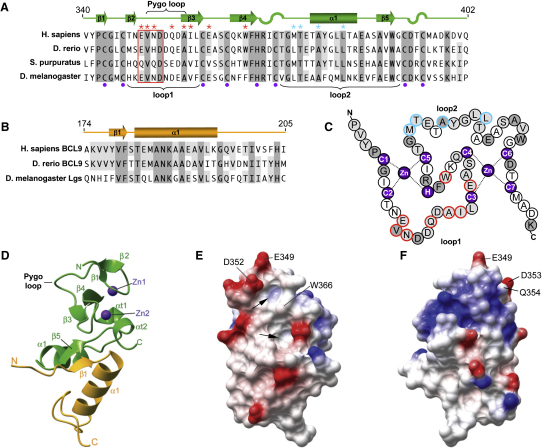
Sequence Alignments and Structure of the Human PHD-HD1 Complex (A and B) Alignments of (A) PHD sequences of hPygo1 (Q9Y3Y4), zebrafish Pygo2 (Q1L8T6), sea urchin Pygo (XP_791313), and *Drosophila* Pygo (Q9V9W8), and (B) HD1 sequences of hBCL9 (O00512), zebrafish BCL9 (Q67FY0), and *Drosophila* Lgs (Q961D9). Dark gray, invariant residues; light gray, semiconserved residues. Marked above sequences are secondary structure elements (β sheets, α helices; α turns are marked by S shapes) and residues involved in HD1 (blue) or H3K4me binding (red) as defined by mutational analysis (Figures 3B and 4E). EVND motif is boxed; indicated are also Zn^2+^-coordinating residues (purple), Pygo loop, and loop1 and loop2 surfaces (brackets). (C) Crossbrace ligation of hPygo1 PHD finger, with Zn-coordinating and mutated residues highlighted (colors as in [A]). (D) Ribbon representation of the hPHD-HD1 complex structure solved at 1.59 Å resolution, with secondary structure elements labeled as in (A) and (B). PHD, green; HD1, orange; Zn^2+^, purple. (E and F) Molecular surface representations of hPHD-HD1, colored according to electrostatic potential (red, negative charges; blue, positive charges), with some Pygo loop and EVND residues labeled. (E) View similar to (D), showing the PHD loop1 surface, with two conspicuous cavities (arrows) separated by W366; (F) is rotated 180° with respect to (E).

To understand how Pygo proteins interact with BCL9, we coexpressed the PHD finger from hPygo1 with its cognate HD1 domain from human BCL9 (Figures 1A and 1B) in bacteria, and purified the resulting complex (hPHD-HD1, the “binary complex”), which was stable over gel filtration and exhibited equimolar binding (see Figure S1 available online). It produced diffracting crystals in several different crystallization conditions, which enabled us to determine its structure by X-ray crystallography at 1.59 Å resolution (Tables 1 and 2).

### The Structure of the Human PHD-HD1 Complex

The overall fold of the Pygo PHD finger, like that of other PHD fingers (Bienz, 2006), forms a compact scaffold that binds two Zn^2+^ ions in a crossbraced fashion (Figures 1A and 1C), as previously shown (Nakamura et al., 2007). It consists of two pairs of antiparallel β strands (whereby β3-β4 is a secondary structural element seen in all known PHD fingers), followed by an α helix and another β strand (β5) in the “loop2” segment (which is unusually large in Pygo proteins; Bienz, 2006), flanked by two α turns (Figures 1A, 1C, and 1D). Note that the “loop1” surface of hPHD contains the highly conserved EVND motif (where V and D are invariant in all known Pygo proteins), which is part of a prominent and flexible surface loop (to be called “Pygo loop,” V350–A356; Figures 1A and 1D).

The HD1 domain of BCL9 is a flat module with a secondary structure that comprises an N-terminal tail, which acquires an extended conformation due to contacts with a symmetry-related molecule, followed by a β strand, an α helix, and an unstructured C-terminal end that folds back toward the N terminus (Figures 1B and 1D).

The binary complex shows a segregation of charges on its molecular surface, with one face being predominantly negatively (Figure 1E), and the opposite positively, charged (Figure 1F). The former presents two conspicuous hydrophobic cavities (Figure 1E, arrows) surrounded by acidic residues and separated by W366, similar to the histone-binding pockets seen in other PHD fingers.

### The Pygo-BCL9 Interface Involves Highly Conserved PHD Loop2 Residues

**Figure 2:**
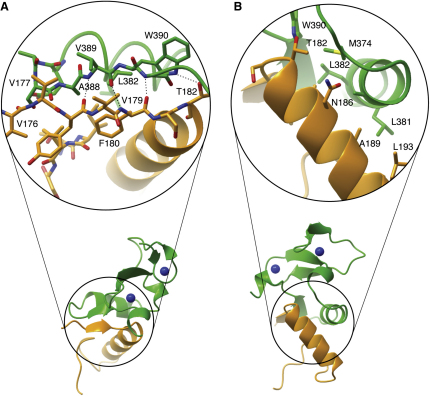
The Pygo-BCL9 Interface Contacts between PHD (green) and HD1 (orange). (A) Parallel β sheets (PHD β5, HD1 β1), (B) α helices. H bonds are indicated by dotted lines; amino acids involved in intermolecular interactions are depicted in cylinder mode.

Two sets of contacts mediate the interaction between hPHD and HD1 (Figure 1D). The first involves primarily β sheet H bonds between PHD β5 (S387, V389, and G391) and HD1 β1 (Y178, V179, and F180) (Figure 2A). These intermolecular interactions depend mainly on backbone atoms. Consistent with this, there is little sequence conservation in this PHD segment, except for A388 (which forms a hydrophobic side-chain interaction with HD1) and W390 (which forms a side-chain interaction with HD1 T182; this tryptophan is also a defining feature of PHD fingers and contributes to their structural cores [Bienz, 2006]).

The second set of contacts comprises a network of hydrophobic side chains, as well as two H bonds (formed by HD1 N186 side chains and PHD backbone atoms; Figure 2B). These side-chain contacts involve either invariant residues (T375, A378, and L382) or a semiconserved PHD residue (M374) (Figure 1A). These were previously mutated to alanine or valine in dPHD, and each individual mutation essentially eliminated Lgs binding; importantly, their combined mutation results in complete inactivation of Pygo, as judged by rescue assays in *pygo* null mutant *Drosophila* embryos (Townsley et al., 2004b). T375A and L382A mutations also block the HD1 binding of mouse Pygo1 (Townsley et al., 2004b). Likewise, the HD1 residues involved in these hydrophobic interactions are either invariant (T182, A185, N186, A189, and I200) or semiconserved (V201; Figure 1B). Systematic N- and C-terminal deletions of HD1 identified V177-I205 as the minimal PHD-interacting fragment (Figure S2), spanning all of these conserved HD1 residues involved in side-chain contacts with PHD.

### Binding of PHD-HD1 Complexes to Methylated Histone H3 Tail Peptides

In vitro pull-down assays with recombinant histones indicated that full-length Pygo proteins (mPygo1, hPygo2, *Drosophila* Pygo) can bind to the histone H3 tail, and that this binding is mediated by their PHD fingers (data not shown; note that the PHD finger is the only domain identifiable in Pygo proteins by BLAST searches). To test whether Pygo PHD fingers exhibit a preference for H3K4me3, like other PHD fingers (see the Introduction), we chose the more quantitative method of isothermal titration calorimetry (ITC) to determine the affinity of bacterially expressed Pygo PHD fingers for various methylated and unmodified 15-mer histone tail peptides.

**Figure 3:**
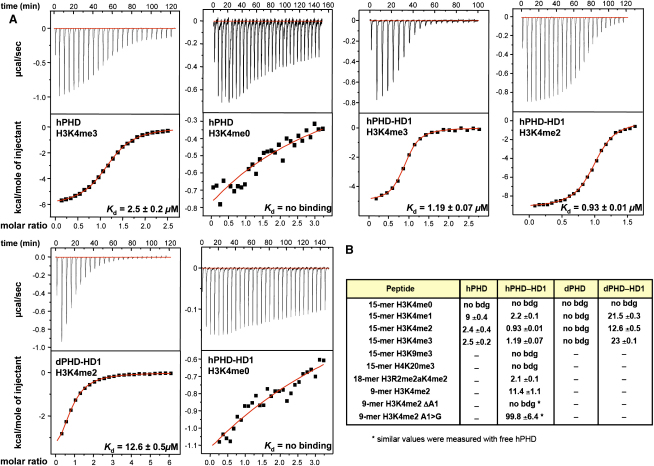
Binding Affinities between Histone Peptides and PHD or PHD-HD1 (A) ITC profiles for the binding of methylated and unmodified 15-mer histone H3 tail peptides to free hPHD, hPHD-HD1, or dPHD-HD1 complex, as indicated in the panels; data were fitted to a one-site model. K_d_ values are given in the individual panels (with fitting errors indicated; see the Supplemental Experimental Procedures). (B) Binding constants (K_d_ values in μM) of free human and *Drosophila* PHD finger versus PHD-HD1 complex for various histone ligands, as indicated.

Indeed, the PHD finger from hPygo1 binds to H3K4me3 and H3K4me2 with similar high affinities (K_d_, 2.5 and 2.4 μM, respectively), and to H3K4me1 with somewhat reduced affinity (K_d_, 9 μM). By contrast, its binding to H3K4me0 was essentially undetectable, like that to other methylated histone tail peptides (H3K9me3 and H4K20me3; Figures 3A and 3B). The PHD finger from hPygo2 also binds to H3K4me3, as shown by preliminary results with NMR spectroscopy (M.F., J.-C. Yang, and D. Neuhaus, unpublished data). However, we were unable to detect H3K4me binding with bacterially expressed dPHD (Figure 3B). Thus, the human, albeit not the *Drosophila*, Pygo PHD fingers recognize specifically H3K4 in its methylated state.

Given that the predicted H3K4me-binding pocket is opposite the HD1-interacting PHD loop2 surface (Figure 1E), we also tested whether PHD fingers could bind to H3K4me peptides after complex formation with HD1. This is the case, and, interestingly, the affinities of the human PHD-HD1 complex for each methylated H3K4 peptide are 2× to 3× higher than that of free hPHD: the highest affinity is seen with H3K4me2 (K_d_, 0.9 μM), but binding to H3K4me3 and H3K4me1 is also in the low micromolar range (K_d_, 1.2 μM and 2.9 μM, respectively) (Figures 3A and 3B).

The effect of PHD-HD1 complex formation on H3K4me binding is even more pronounced in the case of *Drosophila*: recall that we were unable to detect binding of free dPHD to H3K4me; however, the dPHD-HD1 complex shows relatively strong affinities for each methylated H3K4 peptide and, again, a preference for H3K4me2 (K_d_, 13 μM for H3K4me2, 23 μM for H3K4me3, and 22 μM for H3K4me1). Neither complex showed detectable binding to H3K4me0 (Figures 3A and 3B). Therefore, the association of HD1 with human and *Drosophila* PHD fingers enhances their affinities for methylated H3K4 peptides and reveals their modest preference for H3K4me2.

### The Structure of the Ternary Complex

In one of our PHD-HD1 crystals (space group C222_1_, WT2; Table 2), where two binary complexes are seen in the asymmetric unit, one of the N-terminal hPHD ends was inserted into the peptide-binding pocket of the second complex, thus behaving as a pseudoligand (Figure S3). We truncated the N terminus of hPHD by seven residues to avoid this autoinhibition artifact, and crystallized this hPHD-HD1 complex with a 5× molar excess of a 9-mer H3K4me2 peptide (K_d_, 11 μM; Figure 3B), which allowed us to solve the structure of this ternary complex (Tern1) at 1.7 Å resolution (Table 2). We subsequently crystallized the same PHD-HD1 complex with an 18-mer histone H3 tail carrying both K4me2 and asymmetrically methylated R2 (R2me2a) (K_d_, 2.1 μM; Figure 3B), and solved the structure of this alternative ternary complex (Tern2) at 1.60 Å resolution. The latter is virtually identical to the structure of Tern1 (with an rmsd for the Cα backbone of 0.16 Å; Table 2) described below.

**Figure 4:**
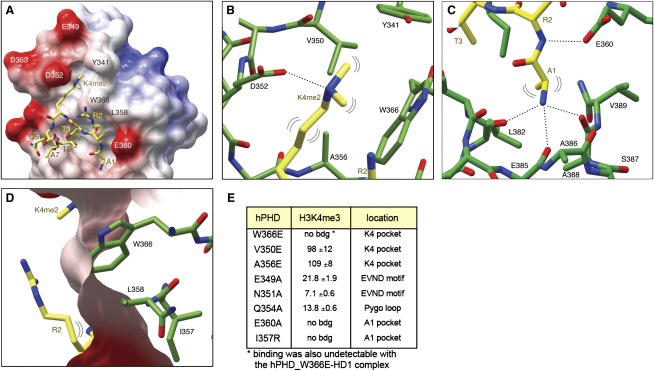
Structures of the Ternary Complex, and H3K4me-Binding Cavities (A) Molecular surface representation of hPHD-HD1 binding to H3K4me2 (in yellow cylinder style), with W366 and other critical residues labeled. (B and C) Cylinder representations of (B) semiaromatic K4me2 cavity and (C) A1 cavity, with critical H bonds indicated as dotted lines and hydrophobic contacts as double brackets. (D) Molecular surface representation of PHD, revealing solvent exposure of R2 (regardless of its methylation status). H3K4me, yellow; PHD cavity residues, green. Note that Tern2 has essentially the same structure as Tern1 (shown here; see text). (E) Binding constants of various hPHD point mutants for H3K4me3 15-mer (K_d_ values in μM; see also Figure 3).

In both maps, we observed additional electron densities in the predicted histone-binding pockets of hPHD, which allowed us to model the first seven amino acids of the histone H3 tail bound to the hPHD-HD1 complex (Figure 4A; Figure S4). The first five amino acids (A1–Q5) exhibit an extended conformation, with R2-K4me2 forming an antiparallel β sheet with PHD β3 (A356–L358) (Figure S5), as observed in all other known PHD-H3K4me structures, while T6 and A7 meander off the PHD surface, looping back on itself, stabilized by an H bond between T6 and T3 (see also Ramon-Maiques et al., 2007). K4me2 and A1 occupy two adjacent cavities, separated by PHD W366 (Figures 1E and 4A).

The K4me2 cavity consists of a semiaromatic cage with W366, Y341, and D352 forming the walls, and V350 and A356 the base (Figure 4B). The two K4me2 methyl groups make hydrophobic interactions with W366 (in PHD β4) and Y341, while D352 (in the EVND motif) forms an H bond with the Nζ of the K4me2 side chain. This H bond could not be formed with K4me3 because the extra methyl group would preclude the approach of the D352 carboxyl group. The K4me2 cavity is different from that in BPTF, ING2/Yng1, and RAG2 that prefer K4me3, but reminiscent of that in the H3K4me0-binding PHD finger of BHC80 (see the Discussion).

The adjacent cavity is deep and accommodates A1 (instead of R2; see below). Specifically, the N-terminal amino group of A1 is tightly bound to backbone carbonyl groups of L382, E385, and A388 (Figure 4C), as in all other PHD fingers. A1 is further stabilized through hydrophobic interactions with L382 and V389 mediated by its Cβ side chain. Notably, all these PHD residues (in α1 or β5) interact simultaneously with HD1 (Figure 2; see also below).

R2 and T3 are located between these two cavities, with the methyl group of T3 buried in a pocket formed by A356, I357, W366, and Y379, and its OH forming an H bond to T383 (Figure S5). Interestingly, although R2 forms two H bonds through its backbone with L358 and with the carboxyl group of the invariant E360, as well as hydrophobic side-chain interactions with L358 and W366 (Figure S5), its charged guanidinium group protrudes from the PHD surface into the solvent (Figure 4D), and adopts multiple conformations in one of the complexes of the asymmetric unit of Tern1. The same is observed in Tern2 (data not shown) in which the methylated guanidinium group of R2 is not able to assume a stable conformation, due to lack of molecular contacts with the PHD finger. This is different from the PHD fingers of BPTF and ING2/Yng1 where R2 is entirely buried in a pronounced pocket adjacent to the K4me cavity. In fact, there is no discernible R2 pocket in the Pygo PHD finger; instead, the long hydrophobic side chain of L358 protrudes into the groove corresponding to the R2 pocket in other PHD fingers, blocking access of R2's side chain and redirecting it into the solvent (Figure 4D). This is somewhat reminiscent of the RAG2 PHD finger, where a tyrosine protrudes into the R2 pocket and interacts with the methyl groups of symmetrically methylated R2 (R2me2s)—though not with R2me2a—thereby stabilizing the conformation of R2 (Ramon-Maiques et al., 2007).

### HD1 Buttresses the Bottom of the PHD A1 Cavity from the Back

Superimposition of our structures of the PHD-HD1 complex on that of the free hPHD finger (Nakamura et al., 2007) shows that the structures are very similar (rmsd for the Cα backbone, 0.6 Å), except for small local conformational changes of two short loop segments.

One of these is the Pygo loop centered on D352, which constitutes one of the walls of the K4me2 pocket (Figure S6A, arrow 1). This loop is flexible, and although its backbone is clearly defined, the electron density for the side chains in some cases is poor. Its conformation varies between the various structures and indeed is different in the two independent molecules of the free PHD (Nakamura et al., 2007). In the ternary complexes, the PHD groups around the K4me2 pocket close up slightly around the peptide (Figure S6B, arrow 1) and the Pygo loop becomes more ordered.

**Figure 5:**
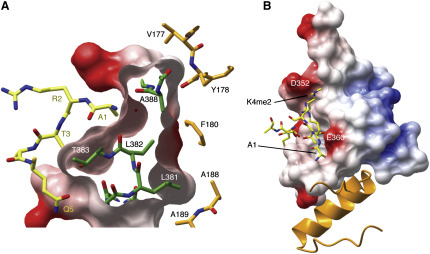
Buttressing of the PHD A1 Cavity by HD1 (A) Molecular surface representation of PHD (green) with electrostatic potential, facing A1 cavity (left, yellow) and HD1 (right, orange). (B) Buttressing of A1 cavity of PHD (molecular surface representation with electrostatic potential) by HD1 (ribbon representation). D352 lip residue of the K4me2 cavity and E360 residue critical for A1 anchoring are indicated.

The second difference is in the PHD-HD1 interface, in a loop segment between α1 and β5 (L382–S387). This loop is pushed out in the binary complex compared to the free PHD finger (Figure S6A, arrow 2), as a direct consequence of the PHD-HD1 interactions (Figure 2), opening up the A1 cavity in the complex. On binding the peptide, there are only small shifts in the residues lining the A1 pocket. Interestingly, these residues that contact A1 are also engaged in contacting HD1, contributing to the PHD-HD1 interface (Figures 2, 4C, and 4D). The base of the A1 cavity is very thin (Figure 5A) and is buttressed from behind by HD1 (Figure 5B). Thus, the PHD-HD1 interactions are likely to shape and stabilize the A1 cavity (see the Discussion).

There is also an apparent conformational change of the HD1 C-terminal tail in the binary versus ternary complex (Figure S6B). However, we believe that the conformation seen in the ternary complex is a crystal artifact, given that there are multiple direct contacts between PHD α1 and β5 and the invariant I200 and semiconserved V201, both included in the minimal PHD-binding fragment of HD1 (Figure S2).

### Function of the H3K4me Pockets in Binding Histone H3 Tail

We conducted ITC binding studies of hPHD with point mutations designed to alter the two cavities, to gain experimental support for their function in H3K4me binding. To test the K4me cavity, we mutated W366 (which forms its proximal wall), and V350 and A356 (which undergo side-chain interactions with K4me2; Figure 4B). No binding whatsoever was detectable with W366E, and V350E and A356E strongly diminished the affinity to H3K4me3, to below 3% of the wild-type (WT) hPHD (Figure 4E). These three residues are therefore critical for ligand recognition. Notably, complex formation of these PHD mutants with HD1 is normal (Figure S1D; data not shown), consistent with our structural data that distinct portions of the PHD-HD1 complex are devoted to the K4 pocket and the HD1-interacting surface, respectively.

Testing the function of the A1 cavity by mutational analysis appeared more challenging because its key residues mostly undergo backbone interactions with the ligand. Nevertheless, we decided to mutate the invariant E360 and I357 that are engaged in direct contacts with A1 and T3, respectively (Figure 4C, Figure S5). Each mutant showed only background levels of H3K4me3 binding (Figure 4E), showing that the structural integrity of the A1 pocket is essential for the binding to the methylated histone H3 tail.

To further confirm the importance of the A1 pocket, we measured the affinities of PHD-HD1 to a truncated H3K4me2 peptide spanning R2-K9 (without A1, ΔA1), and of a more subtly mutated 9-mer whose A1 was substituted with a glycine (A1 > G). No binding was detectable with ΔA1, and the affinity of A1 > G to PHD-HD1 was also reduced to almost background levels (Figure 3B). Evidently, the N-terminal A1 residue of histone H3 is critical for anchoring of its tail in the PHD finger.

Finally, we tested the functional contribution of the Pygo loop to histone binding, by mutating the EVND residues E349 and N351 and the semiconserved Q354 (Figure 4A). The latter is of particular interest because the corresponding residue in *Drosophila* Pygo (D761) proved to be the most critical of a series of individually mutated residues in transient transcription assays in mammalian cells (Townsley et al., 2004b). Indeed, each individual point mutation reduced ligand binding, with the worst affected mutant (E349A) retaining only ∼11% of the WT affinity (K_d_, 22 μM; Figure 4E). These Pygo loop residues thus seem to contribute to ligand binding despite their lack of direct contact with H3K4me2 (Figure 4A).

### An Intact H3K4me-Binding Pocket Is Critical for Pygo Function during *Drosophila* Development

To test whether the binding of Pygo to H3K4me is important for its function in vivo, we conducted rescue assays in *Drosophila* (de la Roche and Bienz, 2007). We introduced a point mutation into V757 (in EVND) of *Drosophila* Pygo, which corresponds to V350 of hPHD that lines its K4me pocket (Figure 4B) and whose mutation drastically reduces its binding to H3K4me3 (Figure 4E). We then generated *pygo* null mutant clones in wing discs, one of the best-characterized Wingless-responsive tissues whose development depends on Pygo function (Belenkaya et al., 2002; de la Roche and Bienz, 2007; Kramps et al., 2002; Parker et al., 2002; Thompson et al., 2002), and we used the GAL4 system to overexpress the Pygo-V757E mutant in these clones, to test its rescue activity in this tissue.

**Figure 6:**
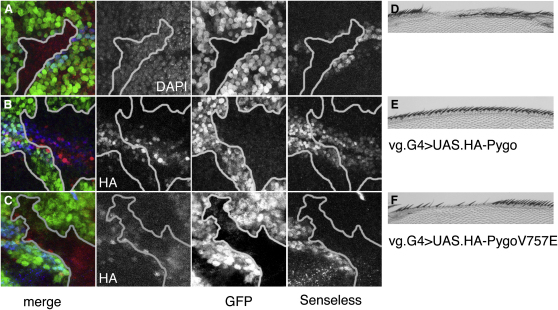
Rescue Assays in *Drosophila*, Revealing Functional Relevance of K4me Cavity (A–C) Wing discs of third instar larvae bearing *pygo^S28^* mutant clones (marked by absence of GFP), with or without overexpressed HA-Pygo, double stained with (A) DAPI (to reveal the nuclei) or (B and C) anti-HA antibody, and (A–C) anti-Senseless antibodies. (D–F) Anterior wing margin of adult flies bearing *pygo^S28^* mutant clones, with or without overexpressed HA-Pygo. (A and D) controls, (B and E) WT HA-Pygo, (C and F) HA-Pygo-V757E.

Overexpression of WT Pygo restored expression of *senseless*, a well-established Wingless target gene in the developing wing discs (de la Roche and Bienz, 2007; Parker et al., 2002), in *pygo* null mutant wing disc clones (Figures 6A and 6B). However, no rescue whatsoever was observed after overexpression of Pygo-V757E in these clones (Figure 6C), although this mutant was expressed at levels comparable to WT Pygo (Figure 6B). Likewise, WT Pygo but not Pygo-V757E rescued all *pygo* mutant phenotypes (i.e., notches and bristle defects) in the anterior wing margin of flies (Figures 6D–6F). We conclude that an intact K4me2-binding pocket is critical for Pygo function during *Drosophila* development.

## Discussion

Our work indicates a physiological role of Pygo proteins in decoding the methylation status of the histone H3 tail, which is critical for the regulation of Wnt-induced transcription. Based on our biochemical and structural evidence, we discuss how Pygo, assisted by BCL9/Lgs as its cofactor, achieves a unique specificity in decoding the combined methylation status at R2 and K4 on the histone H3 tail.

### The Interaction between Pygo and BCL9

Our structures of the PHD-HD1 complex identify the PHD loop2 segment as the BCL9-interacting surface, with a relatively flat and hydrophobic nature. The interaction has two parts, a parallel β sheet between strands PHD β5 and HD1 β1, and a hydrophobic core between the two helices. The buried surface area is relatively small (∼1240 Å^2^, 15% and 18% of the total solvent-accessible surface areas of PHD and HD1, respectively), which is consistent with a relatively transient interaction, as might be expected for a signaling molecule. These structural data are fully consistent with our previous functional analysis during *Drosophila* development (see above; Townsley et al., 2004b).

The structure of the free hPHD finger (Nakamura et al., 2007) shows a dimer in the crystal, in which the PHD-PHD interaction mimics the PHD-HD1 interaction that we see. This PHD-PHD interaction may thus merely reflect a propensity of the hydrophobic loop2 surface to undergo a pseudoligand interaction in the absence of bona fide ligand, similar to the pseudoligand interaction we observed between the hPHD N terminus and the H3K4me-binding pocket (Figure S3). We note that Pygo dimerization is undetectable in vivo (M.F., unpublished data) and so may not be physiologically relevant.

### BCL9 Binding to Pygo Boosts Its Recognition of Methylated Histone H3 Tails

Perhaps our most interesting finding was that the binding of BCL9 HD1 to Pygo PHD increases its affinity to H3K4me. This is particularly striking in *Drosophila* where the H3K4me binding of the Pygo PHD finger depends entirely on its interaction with Lgs HD1 (Figure 3). Given that the backbone structures of human and *Drosophila* PHD-HD1 complexes are very similar (M.F., M.J.S.-B., and M.B., unpublished data), it seems likely that the underlying principle for this boosting effect of HD1 on the H3K4me recognition by PHD is essentially the same in both species.

How does HD1 enhance the H3K4me binding of PHD? The answer is found in the structure of the A1 cavity whose floor is very thin and formed by PHD loop2 residues that are simultaneously engaged in direct interactions with HD1. Indeed, this floor is formed largely by the C-terminal β strand of the PHD finger (Figures 2, 4, and 5A), and although this PHD segment appears to be well structured in the absence of HD1 (Nakamura et al., 2007), this may be partly because the observed PHD-PHD interactions in the crystal mimic the PHD-HD1 contacts. It is therefore possible that the PHD C terminus is somewhat floppy in the absence of HD1, so that its buttressing by HD1 may contribute to the shape and/or stability of the cavity that is required for firm anchoring of the N-terminal alanine residue of the histone H3 tail. Recall that this residue, and the structural integrity of the A1 cavity, are essential for the binding of H3K4me (Figures 3B and 4E).

### Histone H3 Tail Recognition

Recent work indicates that the transition from transcriptional silence to activation of a gene involves a change in the methylation state of (at least) two side chains on the histone H3 tail—R2 and K4 (Guccione et al., 2007; Kirmizis et al., 2007). This is monitored by PHD proteins that bind to this tail, and specific examples that decode the methylation status of K4 have been discovered. Comparison of the structures now available (Lan et al., 2007; Li et al., 2006; Pena et al., 2006; Taverna et al., 2006) suggests how this is done. Namely, the recognition of the H3 tail by PHD fingers depends on two sets of interactions: those that involve a constant H3 segment and provide much of the binding affinity, and those that are sensitive to the methylation status of R2 and K4 and thus provide specificity.

The former set of interactions is similar in all known PHD fingers and comprises (1) the N-terminal alanine of the H3 tail in its deep PHD pocket, which accommodates both the alanine side chain and the terminal NH_3_^+^ group; (2) the T3 side chain, with a hydrophobic pocket and an H bond to recognize the side chain; and (3) the main H3 peptide chain, which forms a short β sheet with the PHD. Recall that the binding of HD1 to Pygo PHD fingers affects primarily this set of interactions, thus boosting the affinity between PHD and histone H3 tail. Other PHD proteins such as BPTF, ING2/Yng1, and BHC80 are subunits of transcription complexes, but it is not known whether similar partner interactions control their binding to modified histone ligands. We note, though, that there are precedents, largely in the context of DNA repair, of different protein modules (e.g., Royal superfamily domains) that cooperate in the binding to modified histone tails (Corsini and Sattler, 2007; Taverna et al., 2007).

Pygo PHD fingers are distinguished from other PHD fingers with regard to the second set of interactions that serve to decode the methylation status of the histone H3 tail. First, they show a modest preference for H3K4me2, which can be explained by the structure of the K4me2 cavity of the PHD-HD1 complex, in particular by the H bond between the pocket lip residue D352 and the side-chain amino group of K4me2 that would not be able to form with K4me3 (Figure 4B). A similar salt bridge with an aspartate residue is observed in the K4me0-binding pocket of BHC80 (Lan et al., 2007), and also in structurally unrelated histone-binding domains with a preference for hypomethylated lysines in various histone tail contexts, such as MBT domains (Grimm et al., 2007; Li et al., 2007b) and the tandem-Tudor domain (Botuyan et al., 2006). By contrast, the PHD fingers with preference for K4me3 contain either a full aromatic cage (BTPF) or semiaromatic, semihydrophobic cages (ING2/Yng1) that provide optimal accommodation of K4me3 through multiple hydrophobic side-chain interactions. Modeling of the K4me3 ligand in the Pygo K4me cavity suggests that this ligand would slightly displace the D352 lip residue in the Pygo loop; however, the high flexibility of this loop would accommodate this. Evidently, such a displacement can be tolerated without much loss of binding affinity (Figure 3), no doubt due to the numerous additional interactions between PHD and histone H3 tail peptide. Especially critical may be the hydrophobic side-chain interaction between one of the K4-methyl groups and the aromatic cavity wall residue W366 (Figure 4B), which is essential for H3K4me binding (Figure 4E), and its interaction with the K4-methyl group(s) may thus be pivotal in discriminating methylated from unmodified H3K4 tails.

The second distinct feature relates to H3R2: in the BPTF and ING2/Yng1 PHD fingers, R2 is completely anchored by its guanidinium group within a groove adjacent to the K4me-binding pocket (Li et al., 2006; Pena et al., 2006; Taverna et al., 2006), which would be incompatible with methylation. Indeed, the H3K4me binding of the SET1 PHD finger is blocked by R2me2a (Kirmizis et al., 2007). However, in both our ternary complexes, only the first two carbons of the R2 side chain are fixed (Figure 4C), while the guanidinium group at the end of this side chain is pushed out into the solvent by the side chain of L358 (Figure 4D), regardless of the methylation status of R2. Taken together with our binding data that revealed insensitivity to R2 methylation, this implies that Pygo PHD fingers recognize equally well histone H3 tails that are methylated or unmodified at R2. Interestingly, R2me2a appears to be linked to transcriptional silence, and is mutually exclusive with H3K4me3 in chromatin (Guccione et al., 2007; Kirmizis et al., 2007), which itself is a histone mark for gene activity (Kouzarides, 2007).

These two unique structural features of Pygo's H3K4me-binding pocket enable it to recognize histone H3 tails that bear both K4me2 and R2me2a marks. The combined occurrence of these two chromatin marks is indicative of an early step in the transition from gene silence to gene activation: evidence suggests that, for a gene to become fully active, H3R2me2a needs to be demethylated before H3K4 can be trimethylated by SET1 methyltransferases (Guccione et al., 2007; Kirmizis et al., 2007).

Based on our structural analysis, we propose that Pygo plays a role early during the transition from gene silence to Wnt-induced gene activation (e.g., in step 1 of the proposed sequence of events; Kirmizis et al., 2007). Indeed, the Pygo-BCL9 complex may function to expose H3R2me2a for demethylation (by an as-yet-unknown enzyme), prior to recruiting the SET1 methyltransferase complex (Sierra et al., 2006) through capturing β-catenin at TCF target genes during incipient Wnt signaling (de la Roche and Bienz, 2007; Townsley et al., 2004a). SET1 recruitment would then result in the conversion of H3K4me2 to H3K4me3, which in turn would allow full transcriptional activity of TCF target genes during sustained Wnt signaling.

### The Potential of the Pygo H3K4me-Binding Pockets as Targets for Small-Molecule Inhibitors

Despite the importance of the Wnt pathway in cancer, there are no well-established small-molecule inhibitors of this pathway to date, probably because the oncogenic mutations that cause pathway hyperactivation typically occur at the level of, or immediately above, β-catenin (Bienz and Clevers, 2000; Polakis, 2000). However, there are no enzymes to inhibit below activated β-catenin, and the protein interaction surface between β-catenin and TCF is highly unsuitable as a drug target due to its extensive nature and multiple overlapping interactions with negative regulators of the Wnt pathway (Daniels et al., 2001).

The function of the Pygo-BCL9 complex in Wnt signaling depends on at least three physiologically relevant protein-protein interactions: those between PHD and its two ligands (HD1 and histone H3 tail) described here, and that between BCL9 HD2 and β-catenin (Sampietro et al., 2006). Each of these is known at the structural level and is sensitive to point mutations of single residues (Sampietro et al., 2006; Townsley et al., 2004b; this work), suggesting that they may have potential as targets for disruption by small compounds.

## Experimental Procedures

### Expression and Purification of Proteins, and ITC Measurements

hPygo1 PHD (amino acids 333–402) or *Drosophila* Pygo PHD (amino acids 747–808) fused to GST, and hBCL9 HD1 (amino acids 177–205) or Lgs HD1 (amino acids 316–350) fused to MBP, were coexpressed in a bicistronic expression vector (including N-terminal TEV protease sites for removal of tags) in *E*. *coli* BL21-CodonPlus(DE3)-RIL cells (Stratagene), and PHD-HD1 complexes were purified as described in the Supplemental Experimental Procedures (see also Figure S1). Free PHD fingers were purified similarly to the PHD-HD1 complexes, and purity was assessed by SDS-PAGE prior to use for ITC. ITC was performed after dialysis of protein into 100 mM NaCl, 25 mM Tris-HCl (pH 8.0), 10 μM ZnCl_2_, and 2 mM β-mercaptoethanol, essentially as described (Grimm et al., 2007) (see also the Supplemental Experimental Procedures).

### Crystallization

Concentrated protein was centrifuged at 100,000 × g for 15 min and used for crystallization (initial screen of 1500 different crystallization conditions in a 96-well sitting drop format using 100 nl drops; Stock et al., 2005). Crystals grown at 19°C by the vapor diffusion method emerged after ∼24 hr under multiple conditions. The following conditions were used: 1.7 M (NH_4_)_2_SO_4_, 100 mM Tris (pH 7.5), and 200 mM NaCl (for hPHD_W366F-HD1); 1 M Na-citrate, 100 mM Tris (pH 7), and 200 mM NaCl (for WT1); and 1.3 M (NH_4_)_2_SO_4_, 100 mM Tris (pH 6.5), and 200 mM NaCl (for WT2, space group C222_1_). For the ternary complexes, 10 mg/ml of hPHD-HD1 was mixed with 5× molar excess of H3K4me2 9-mer (Figure 4) or H3R2me2aK4me2 18-mer peptide (1 hr incubation on ice followed 15 min centrifugation at 100,000 × g) prior to crystallization with 26.4% PEG-3350, 200 mM LiSO_4_, and 100 mM Tris (pH 7), or 20% PEG-4000, 20% isopropanol, and 0.1 M Na citrate, respectively; for both complexes, amino acids 340–402 of hPHD were used, to avoid blocking of the H3K4me2 pocket (Figure S3). Each crystal was soaked for < 1 min in 25% glycerol as cryoprotectant before flash-cooling in liquid nitrogen.

### Diffraction Data Collection and Structure Solution

Different diffraction data sets were collected at ESRF and Diamond synchrotrons (Tables 1 and 2). The initial hPHD-HD1 structure was phased using the anomalous signal from the Zn^2+^ ions binding to PHD. A fluorescence scan was performed to select the wavelengths and collect a MAD data set from a crystal of hPHD_W366F-HD1 (Table 1); the peak was collected first, followed by the inflexion point and the remote. Crystallographic data were processed, and the structure was phased, as described in the supplement. The initial structure of hPHD_W366F-HD1 was used to solve four other crystal forms by molecular replacement with Phaser (Table 2) (McCoy et al., 2005): WT1 (isomorphous with hPHD_W366F-HD1) at higher resolution; WT2, a second crystal form (space group C222_1_) exhibiting pseudoligand binding (Figure S3); and the two ternary complexes.

### Rescue Assays in *Drosophila*

HA-tagged Pygo-V757E was constructed in pUAST, and multiple independent transformant lines were isolated, as described previously for WT HA-Pygo (Townsley et al., 2004b). Two different lines were each tested for rescue activity of *pygo* null mutant clones in the wing disc, as described (de la Roche and Bienz, 2007).

## Figures and Tables

**Table 1:** Data Collection, Phasing, and Refinement Statistics

hPHD_W366F-HD1	Peak (P)	Inflexion (Ip)		Remote (Rm)
Beamline	ESRF, ID23-1			
Strategy	180°, Δφ 1°	180°, Δφ 1°		180°, Δφ 1°
Wavelength	1.2832	1.2841		1.0000
Space Group	I222			
a, b, c (Å)	32.56, 71.91, 91.56			
α, β, γ (°)	90.0, 90.0, 90.0			
Resolution (Å)	30.67–1.96 (2.07–1.96)^a^	35.97–1.96 (2.07–1.96)		24.96–1.65 (1.74–1.65)
R_merge_ (%)^b^	4.6 (11.6)	4.7 (13.8)		5.3 (38.0)
I/σ(I)	30.7 (13.8)	29.1 (11.5)		21.7 (4.7)
Completeness (%)	99.9 (99.9)	99.9 (99.9)		99.6 (99.6)
Multiplicity	6.8 (6.7)	6.8 (6.7)		6.8 (7.0)
Anomalous Completeness (%)	99.8 (99.6)	99.8 (99.5)		99.5 (100)
Anomalous Multiplicity	3.6 (3.5)	3.6 (3.5)		3.6 (3.6)
Complexes in A.U.				1

Phasing	N Acentric (A)	FOM Acentric	N Centric (C)	FOM centric

Overall	11,649	0.57	1707	0.46
Phasing power	Isomorphous (A)	Isomorphous (C)	Anomalous (A)	
Pk	0.0	0.0	3.4	
Ip	2.5	1.7	2.2	
Rm	1.7	1.1	1.4	

Refinement				

Resolution (Å)				24.96–1.65 (1.69–1.65)
Number of reflections				12,608
Test set size (%)				5.0
R_work_ (%)				21.7 (25.8)
R_free_ (%)				26.7 (29.0)
Number of atoms (non-H)				885
Residues (PHD/HD1)				333–397/174–205
<B> (Å^2^)				28.7
Rmsd				
Bond length (Å)				0.012
Bond angle (°)				1.372
Ramachandran plot				
In favored regions (%)				98.0
In allowed regions (%)				2.0
Outliers (%)				0.0

a Highest resolution shell (in Å) shown in parentheses.

b R_merge_ = Σ_hkl_ |I_hkl_ − <I_hkl_>|/Σ_hkl_ I_hkl_.

**Table 2:** Data Collection and Refinement Statistics

Crystal	WT1 Binary	WT2 Binary	Tern1	Tern2
Beamline	ESRF, ID14-1	ESRF, ID14-3	Diamond, I03	ESRF, ID14-1
Strategy	225°, Δφ 0.5°	180°, Δφ 0.5°	135°, Δφ 0.5°	90°, Δφ 0.5°
Wavelength (Å)	0.934	0.931	1.06	0.934
Space Group	I222	C222_1_	P42_1_2	P42_1_2
a, b, c (Å)	32.10, 79.47, 91.83	88.72, 100.89, 64.24	105.72, 105.72, 51.43	105.75, 105.75, 51.42
α, β, γ (°)	90, 90, 90	90, 90, 90	90, 90, 90	90, 90, 90
Resolution (Å)	28.56–1.59 (1.68–1.59)^a^	31.450–2.77 (2.92–2.77)	42.37–1.70 (1.79–1.70)	26.44–1.6 (1.69–1.60)
R_merge_ (%)^b^	5.6 (37.4)	6.3 (37.7)	11.8 (121.6)	11.7 (60.1)
I/σ(I)	26.6 (11.4)	22.5 (4.2)	12.4 (2.2)	11.4 (3.1)
Completeness (%)	99.9 (99.9)	99.7 (99.7)	100 (100)	100 (100)
Multiplicity	10.5 (10.3)	7.0 (6.9)	10.3 (10.2)	7.1 (7.0)
Complexes in A.U.	1	2	2	2

Refinement				

Resolution (Å)	28.56–1.59 (1.63–1.59)	31.450–2.77 (2.84–2.77)	42.37–1.70 (1.79–1.70)	26.44–1.60 (1.64–1.60)
Number of reflections	15,426	7263	30,994	37,079
Test set size (%)	5.0	4.6	5.1	5.0
R_work_ (%)	19.2 (18.6)	23.3 (29.5)	19.3 (29.5)	20.1 (27.2)
R_free_ (%)	22.5 (19.6)	26.7 (42.4)	23.0 (38.1)	21.8 (32.1)
Number of atoms (non-H)	812	1473	1688	1759
Residues (PHD/HD1)	341–397/173–205	333–397/174–205	339–398/174–203	339–398/174–203
<B> (Å^2^)	22.9	64.8	28.8	22.0
Rmsd				
Bond length (Å)	0.010	0.008	0.011	0.010
Bond angle (°)	1.147	0.986	1.197	1.210
Ramachandran plot				
In favored regions (%)	97.8	94.1	97.8	97.2
In allowed regions (%)	1.1	4.8	2.2	2.8
Outliers (%)	1.1 (S362^c^)	1.1 (S362^c^)	0.0	0.0

a Highest resolution shell (in Å) shown in parentheses.

b R_merge_ = Σ_hkl_ |I_hkl_ − <I_hkl_>|/Σ_hkl_ I_hkl_.

c S362 presents an unusual conformation (the electron density map is clear in this area) because it is next to the C363, which is part of the second Zn^2+^-binding site (Zn2).

## Accession Numbers

Coordinates and structure factors have been deposited with the Protein Data Bank under accession codes 2vp7 (hPHD_W366F-HD1), 2vpb (WT1 Binary), 2vpd (WT2 Binary), 2vpe (Tern1), and 2vpg (Tern2).

## Supplemental Data

Document S1. Supplemental Experimental Procedures, Supplemental References, and Six Figures

## References

[bib1] Adachi S., Jigami T., Yasui T., Nakano T., Ohwada S., Omori Y., Sugano S., Ohkawara B., Shibuya H., Nakamura T., Akiyama T. (2004). Role of a BCL9-related β-catenin-binding protein, B9L, in tumorigenesis induced by aberrant activation of Wnt signaling. Cancer Res..

[bib2] Arce L., Yokoyama N.N., Waterman M.L. (2006). Diversity of LEF/TCF action in development and disease. Oncogene.

[bib3] Belenkaya T.Y., Han C., Standley H.J., Lin X., Houston D.W., Heasman J., Lin X. (2002). pygopus encodes a nuclear protein essential for wingless/Wnt signaling. Development.

[bib4] Bienz M. (2006). The PHD finger, a nuclear protein-interaction domain. Trends Biochem. Sci..

[bib5] Bienz M., Clevers H. (2000). Linking colorectal cancer to Wnt signaling. Cell.

[bib6] Botuyan M.V., Lee J., Ward I.M., Kim J.E., Thompson J.R., Chen J., Mer G. (2006). Structural basis for the methylation state-specific recognition of histone H4-K20 by 53BP1 and Crb2 in DNA repair. Cell.

[bib7] Brembeck F.H., Schwarz-Romond T., Bakkers J., Wilhelm S., Hammerschmidt M., Birchmeier W. (2004). Essential role of BCL9-2 in the switch between β-catenin's adhesive and transcriptional functions. Genes Dev..

[bib8] Clevers H. (2006). Wnt/β-catenin signaling in development and disease. Cell.

[bib9] Corsini L., Sattler M. (2007). Tudor hooks up with DNA repair. Nat. Struct. Mol. Biol..

[bib10] Daniels D.L., Eklof Spink K., Weis W.I. (2001). β-catenin: molecular plasticity and drug design. Trends Biochem. Sci..

[bib11] de la Roche M., Bienz M. (2007). Wingless-independent association of Pygopus with dTCF target genes. Curr. Biol..

[bib12] Grimm C., Gaytan de Ayala Alonso A., Rybin V., Steuerwald U., Ly-Hartig N., Fischle W., Müller J., Müller C.W. (2007). Structural and functional analyses of methyl-lysine binding by the malignant brain tumour repeat protein Sex comb on midleg. EMBO Rep..

[bib13] Guccione E., Bassi C., Casadio F., Martinato F., Cesaroni M., Schuchlautz H., Luscher B., Amati B. (2007). Methylation of histone H3R2 by PRMT6 and H3K4 by an MLL complex are mutually exclusive. Nature.

[bib14] Hoffmans R., Basler K. (2004). Identification and in vivo role of the Armadillo-Legless interaction. Development.

[bib15] Hoffmans R., Städeli R., Basler K. (2005). Pygopus and legless provide essential transcriptional coactivator functions to armadillo/β-catenin. Curr. Biol..

[bib16] Jenuwein T., Allis C.D. (2001). Translating the histone code. Science.

[bib17] Kirmizis A., Santos-Rosa H., Penkett C.J., Singer M.A., Vermeulen M., Mann M., Bahler J., Green R.D., Kouzarides T. (2007). Arginine methylation at histone H3R2 controls deposition of H3K4 trimethylation. Nature.

[bib18] Kouzarides T. (2007). Chromatin modifications and their function. Cell.

[bib19] Kramps T., Peter O., Brunner E., Nellen D., Froesch B., Chatterjee S., Murone M., Züllig S., Basler K. (2002). Wnt/wingless signaling requires BCL9/legless-mediated recruitment of pygopus to the nuclear β-catenin-TCF complex. Cell.

[bib20] Lake B.B., Kao K.R. (2003). Pygopus is required for embryonic brain patterning in Xenopus. Dev. Biol..

[bib21] Lan F., Collins R.E., De Cegli R., Alpatov R., Horton J.R., Shi X., Gozani O., Cheng X., Shi Y. (2007). Recognition of unmethylated histone H3 lysine 4 links BHC80 to LSD1-mediated gene repression. Nature.

[bib22] Li H., Ilin S., Wang W., Duncan E.M., Wysocka J., Allis C.D., Patel D.J. (2006). Molecular basis for site-specific read-out of histone H3K4me3 by the BPTF PHD finger of NURF. Nature.

[bib23] Li B., Rheaume C., Teng A., Bilanchone V., Munguia J.E., Hu M., Jessen S., Piccolo S., Waterman M.L., Dai X. (2007). Developmental phenotypes and reduced Wnt signaling in mice deficient for pygopus 2. Genesis.

[bib24] Li H., Fischle W., Wang W., Duncan E.M., Liang L., Murakami-Ishibe S., Allis C.D., Patel D.J. (2007). Structural basis for lower lysine methylation state-specific readout by MBT repeats of L3MBTL1 and an engineered PHD finger. Mol. Cell.

[bib25] McCoy A.J., Grosse-Kunstleve R.W., Storoni L.C., Read R.J. (2005). Likelihood-enhanced fast translation functions. Acta Crystallogr. D Biol. Crystallogr..

[bib26] Nakamura Y., Umehara T., Hamana H., Hayashizaki Y., Inoue M., Kigawa T., Shirouzu M., Terada T., Tanaka A., Padmanabhan B., Yokoyama S. (2007). Crystal structure analysis of the PHD domain of the transcription co-activator Pygopus. J. Mol. Biol..

[bib27] Parker D.S., Jemison J., Cadigan K.M. (2002). Pygopus, a nuclear PHD-finger protein required for Wingless signaling in Drosophila. Development.

[bib28] Pena P.V., Davrazou F., Shi X., Walter K.L., Verkhusha V.V., Gozani O., Zhao R., Kutateladze T.G. (2006). Molecular mechanism of histone H3K4me3 recognition by plant homeodomain of ING2. Nature.

[bib29] Polakis P. (2000). Wnt signaling and cancer. Genes Dev..

[bib30] Ramon-Maiques S., Kuo A.J., Carney D., Matthews A.G., Oettinger M.A., Gozani O., Yang W. (2007). The plant homeodomain finger of RAG2 recognizes histone H3 methylated at both lysine-4 and arginine-2. Proc. Natl. Acad. Sci. USA.

[bib31] Sampietro J., Dahlberg C.L., Cho U.S., Hinds T.R., Kimelman D., Xu W. (2006). Crystal structure of a β-catenin/BCL9/Tcf4 complex. Mol. Cell.

[bib32] Schwab K.R., Patterson L.T., Hartman H.A., Song N., Lang R.A., Lin X., Potter S.S. (2007). Pygo1 and Pygo2 roles in Wnt signaling in mammalian kidney development. BMC Biol..

[bib33] Shi X., Hong T., Walter K.L., Ewalt M., Michishita E., Hung T., Carney D., Pena P., Lan F., Kaadige M.R. (2006). ING2 PHD domain links histone H3 lysine 4 methylation to active gene repression. Nature.

[bib34] Shi X., Kachirskaia I., Walter K.L., Kuo J.H., Lake A., Davrazou F., Chan S.M., Martin D.G., Fingerman I.M., Briggs S.D. (2007). Proteome-wide analysis in Saccharomyces cerevisiae identifies several PHD fingers as novel direct and selective binding modules of histone H3 methylated at either lysine 4 or lysine 36. J. Biol. Chem..

[bib35] Sierra J., Yoshida T., Joazeiro C.A., Jones K.A. (2006). The APC tumor suppressor counteracts β-catenin activation and H3K4 methylation at Wnt target genes. Genes Dev..

[bib36] Song N., Schwab K.R., Patterson L.T., Yamaguchi T., Lin X., Potter S.S., Lang R.A. (2007). pygopus 2 has a crucial, Wnt pathway-independent function in lens induction. Development.

[bib37] Städeli R., Basler K. (2005). Dissecting nuclear Wingless signalling: recruitment of the transcriptional co-activator Pygopus by a chain of adaptor proteins. Mech. Dev..

[bib38] Stock D., Perisic O., Löwe J. (2005). Robotic nanolitre protein crystallisation at the MRC Laboratory of Molecular Biology. Prog. Biophys. Mol. Biol..

[bib39] Taverna S.D., Ilin S., Rogers R.S., Tanny J.C., Lavender H., Li H., Baker L., Boyle J., Blair L.P., Chait B.T. (2006). Yng1 PHD finger binding to H3 trimethylated at K4 promotes NuA3 HAT activity at K14 of H3 and transcription at a subset of targeted ORFs. Mol. Cell.

[bib40] Taverna S.D., Li H., Ruthenburg A.J., Allis C.D., Patel D.J. (2007). How chromatin-binding modules interpret histone modifications: lessons from professional pocket pickers. Nat. Struct. Mol. Biol..

[bib41] Thompson B.J. (2004). A complex of Armadillo, Legless, and Pygopus coactivates dTCF to activate wingless target genes. Curr. Biol..

[bib42] Thompson B., Townsley F., Rosin-Arbesfeld R., Musisi H., Bienz M. (2002). A new nuclear component of the Wnt signalling pathway. Nat. Cell Biol..

[bib43] Townsley F.M., Cliffe A., Bienz M. (2004). Pygopus and Legless target Armadillo/β-catenin to the nucleus to enable its transcriptional co-activator function. Nat. Cell Biol..

[bib44] Townsley F.M., Thompson B., Bienz M. (2004). Pygopus residues required for its binding to Legless are critical for transcription and development. J. Biol. Chem..

[bib45] Willert K., Jones K.A. (2006). Wnt signaling: is the party in the nucleus?. Genes Dev..

[bib46] Wysocka J., Swigut T., Xiao H., Milne T.A., Kwon S.Y., Landry J., Kauer M., Tackett A.J., Chait B.T., Badenhorst P. (2006). A PHD finger of NURF couples histone H3 lysine 4 trimethylation with chromatin remodelling. Nature.

