# Supplementary material for: Decoding of Methylated Histone H3 Tail by the Pygo-BCL9 Wnt Signaling Complex
Source: Mol Cell. 2008 May 23;30(4-2):507–18. doi: 10.1016/j.molcel.2008.03.011 (PMC2726290; doi:10.1016/j.molcel.2008.03.011)
Supplement: Document S1. Supplemental Experimental Procedures, Supplemental References, and Six Figures [file mmc1.pdf]

Molecular Cell, Volume *30*

Supplemental Data

Decoding of Methylated Histone H3 Tail by the Pygo-BCL9 Wnt Signaling Complex

Marc Fiedler, María José Sánchez-Barrena, Maxim Nekrasov, Juliusz Mieszczanek, Vladimir Rybin, Jürg Müller, Phil Evans, and Mariann Bienz

Supplemental Experimental Procedures

E. coli BL21-CodonPlus(DE3)-RIL cells (Stratagene) co-expressing hPHD and hHD1 were grown at 37°C in LB to OD_{600} of 0.4, reducing the temperature to 24°C followed by induction with 1mM IPTG at OD_{600} of 0.7. After 3-5 h induction, cells were pelleted, shock-frozen in liquid nitrogen and stored at –70°C until needed. Cell pellets were re-suspended in PBS supplemented with 1mM DTT and complete EDTA-free protease inhibitor cocktail tablets (Roche), lyzed by passing twice through an EmulsiFlex-C5 (Avestin) and centrifuged at 100,000 *g* for 30 min. The supernatant was incubated with Glutathione Sepharose 4B resin (Amersham Biosciences). Resin was washed extensively using PBS with increasing concentrations of NaCl (300mM, 600mM, 900mM). Both N-terminal tags of the equimolar protein complex were removed by TEV protease (1:80) cleavage O/N at 4°C. The resulting PHD-HD1 complex was separated from GST and MBP by gel filtration on a HiPrep26/60 G75 (GE Healthcare). Fractions containing the complex were concentrated and separated once more by gel filtration. The gel filtration buffer (25mM Tris pH8.0, 200mM NaCl, 5mM β-ME, 50μM $ZnCl_2$, 0.06% NaN_3) containing the pure protein complex was exchanged to 5mM Tris pH8.0, 25mM NaCl, 1mM DTT and 0.01% NaN_3 and concentrated to 10mg/ml using a Vivaspin 20 concentrator (3kD MWCO, Vivascience).

ITC was carried out using a VP-ITC Microcal calorimeter (Microcal, Northhampton, MA, USA). Peptides were lyophilized against freshly prepared 20mM ammonium bicarbonate solution and resuspended in the ITC dialysis buffer (25mM Tris pH 8.0, 100mM NaCl and 2mM β-mercaptoethanol). Experiments were performed at 25°C. During titration, 4-12μl aliquots of 0.5-1.0mM peptide solution was injected into a solution of 50μM protein at time intervals of 5 min. ITC data were corrected for the heat of dilution and analyzed using Origin (version 5.0) provided by the manufacturer.

Crystallographic data were processed using Mosflm (Leslie, 2006) and scaled with Scala (Evans, 2006). Using the MAD data, two Zn^{2+} ions were found with auto-SHARP (Bricogne et al., 2003). Experimental phases were improved by solvent flattening with Solomon (Abrahams and Leslie, 1996) using a solvent content of 35.2%. The experimental map was of excellent quality, and 85% of the final model was built automatically with ARP/wARP (Lamzin et al., 2001). All structures were refined using Refmac (Murshudov et al., 1997), and the models were updated using Coot (Emsley and Cowtan, 2004). The stereochemistry of the models was verified with MolProbity (Davis et al., 2007). Secondary structure elements were assigned by the method of Kabsch and Sanders (Kabsch and Sanders, 1983) using CCP4mg (Potterton et al., 2004). Analysis of structures was done with CCP4i programs (Potterton et al., 2003), and images were drawn with CCP4mg (Potterton et al., 2004).

Supplemental References

Abrahams, J. P., and Leslie, A. G. (1996). Methods used in the structure determination of bovine mitochondrial F1 ATPase. Acta Crystallogr D Biol Crystallogr *52*, 30-42.

Bricogne, G., Vonrhein, C., Flensburg, C., Schiltz, M., and Paciorek, W. (2003). Generation, representation and flow of phase information in structure determination: recent developments in and around SHARP 2.0. Acta Crystallogr D Biol Crystallogr *59*, 2023-2030.

Davis, I. W., Leaver-Fay, A., Chen, V. B., Block, J. N., Kapral, G. J., Wang, X., Murray, L. W., Arendall, W. B., 3rd, Snoeyink, J., Richardson, J. S., and Richardson, D. C. (2007). MolProbity: all-atom contacts and structure validation for proteins and nucleic acids. Nucleic Acids Res *35*, W375-383.

Emsley, P., and Cowtan, K. (2004). Coot: model-building tools for molecular graphics. Acta Crystallogr D Biol Crystallogr *60*, 2126-2132.

Evans, P. (2006). Scaling and assessment of data quality. Acta Crystallogr D Biol Crystallogr *62*, 72-82.

Kabsch, W., and Sanders, C. (1983). Dictionary of protein secondary structure - pattern recognition of hydrogen-bonded and geometrical features. Biopolymers *22*, 2577-2637.

Lamzin, V. S., Perrakis, A., and Wilson, K. S. (2001). International Tables for Crystallography: Crystallography of Biological Macromolecules, 720. *Rossmann, M. Arnold, E. Editors*, Kluwer Academic, Dordrecht, The Netherlands.

Leslie, A. G. (2006). The integration of macromolecular diffraction data. Acta Crystallogr D Biol Crystallogr *62*, 48-57.

Murshudov, G. N., Vagin, A. A., and Dodson, E. J. (1997). Refinement of macromolecular structures by the maximum-likelihood method. Acta Crystallogr D Biol Crystallogr *53*, 240-255.

Nakamura, Y., Umehara, T., Hamana, H., Hayashizaki, Y., Inoue, M., Kigawa, T., Shirouzu, M., Terada, T., Tanaka, A., Padmanabhan, B., and Yokoyama, S. (2007). Crystal structure analysis of the PHD domain of the transcription co-activator Pygopus. J Mol Biol *370*, 80-92.

Potterton, E., Briggs, P., Turkenburg, M., and Dodson, E. (2003). A graphical user interface to the CCP4 program suite. Acta Crystallogr D Biol Crystallogr *59*, 1131-1137.

Potterton, L., McNicholas, S., Krissinel, E., Gruber, J., Cowtan, K., Emsley, P., Murshudov, G. N., Cohen, S., Perrakis, A., and Noble, M. (2004). Developments in the CCP4 molecular-graphics project. Acta Crystallogr D Biol Crystallogr *60*, 2288-2294.

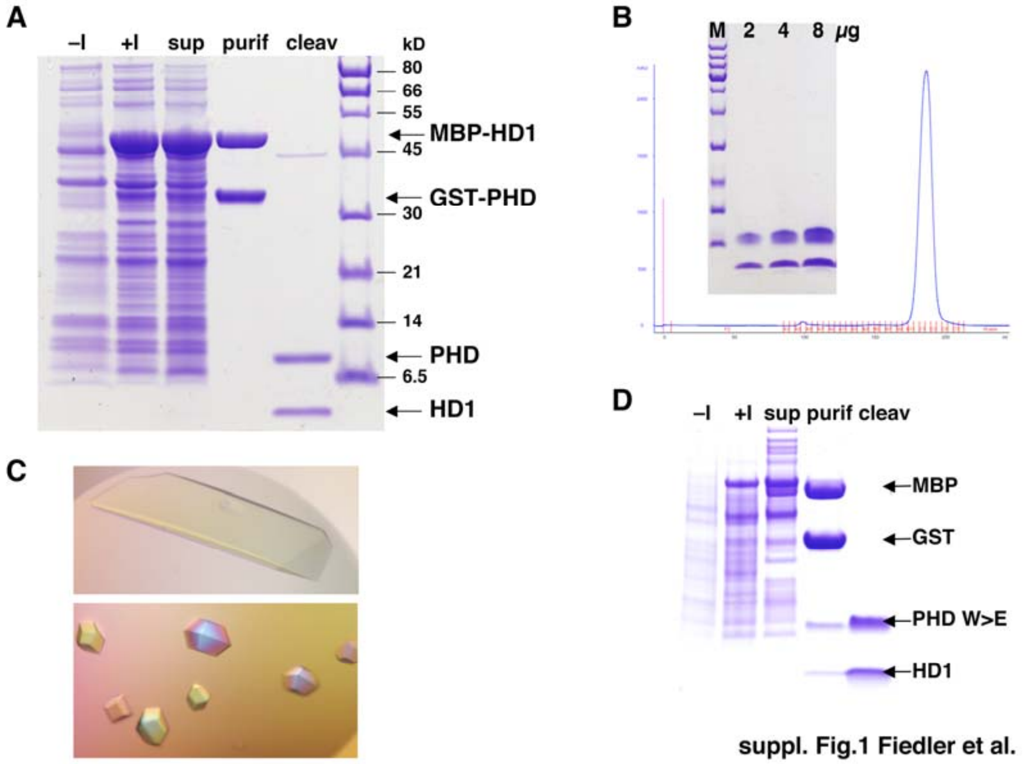

Figure S1. Expression, Purification, and Crystallization of WT and Mutant hPHD-HD1

(A) SDS-PAGE of total lysates from bacteria expressing WT hPHD-HD1 complex (–I uninduced; +I after induction). Soluble protein (sup) was purified via GST affinity resin yielding the pure, equimolar complex (purif). The protein tags were cleaved off by TEV protease and removed by gel filtration, resulting in pure PHD-HD1 complex (cleav).

(B) The PHD-HD1 complex was stable over gel filtration and eluted in one peak. SDS-PAGE revealed purity and equimolar binding of the partners.

(C) Examples of crystals from pure hPHD-HD1 complex crystallized under different conditions.

(D) SDS-PAGE of total bacterial lysates, as in (A), showing normal yields of mutant hPHD_W366E-HD1 complex. Complex formation with HD1 was also normal in the case of other mutant PHD fingers whose K4me-binding pockets are defective, such as PHD_V350E (not shown).

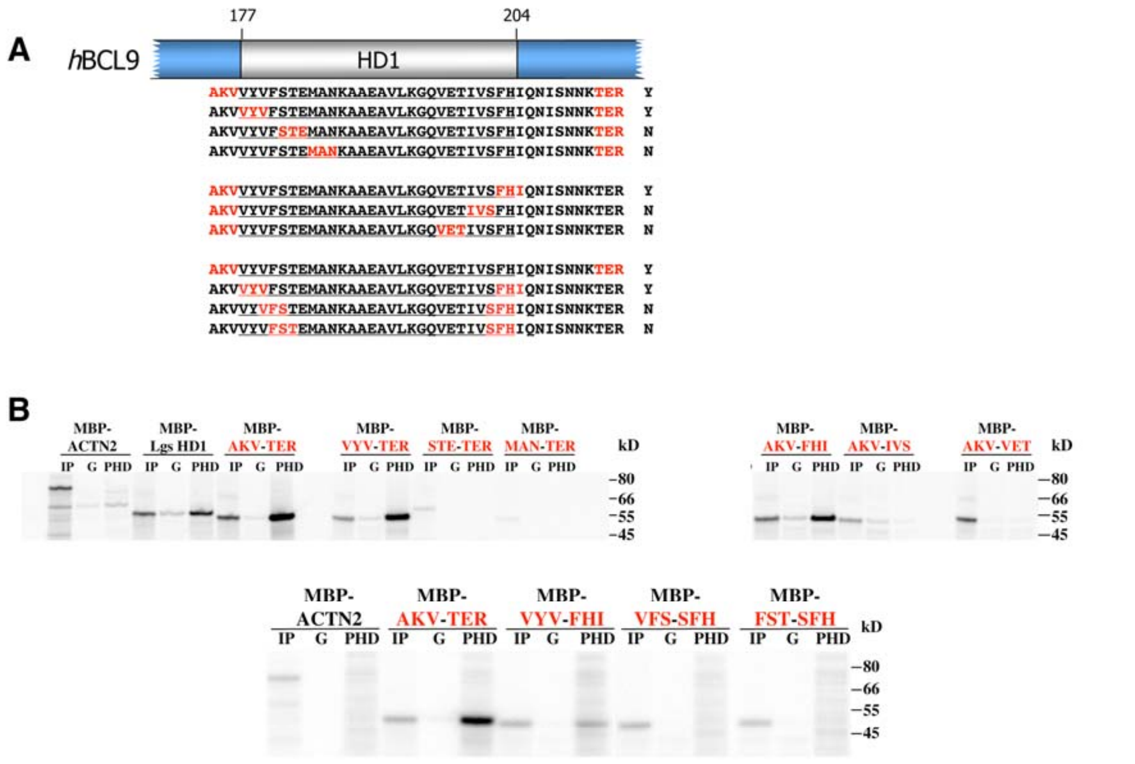

suppl. Fig.2 Fiedler et al.

Figure S2. Deletion Analysis of hBCL9 HD1 and Binding to hPygo1 PHD

(A) Systematic N- and C-terminal deletions of HD1 from hBCL9 (underlined), individually and combined (bottom); sequences between, and including, red (marking deletion end-points) were tested for their *in vitro* binding to PHD from hPygo1 (B), as summarized on the right; Y, binding; N, no binding.

(B) *In vitro* pull-down assays, to test binding between MBP-tagged HD1 deletions and GST-hPHD; positive controls, Lgs HD1, hBCL9 AKV-TER; negative controls, GST, MBP-ACTN2; IP, input (5%); G, GST; PHD, GST-hPHD.

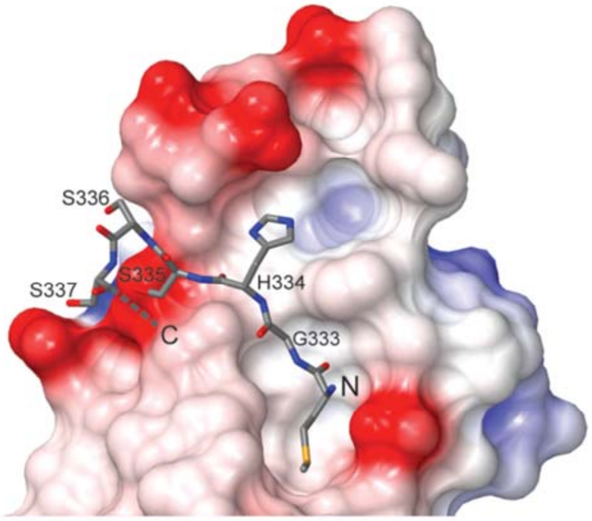

suppl. Fig.3 Fiedler et al.

Figure S3. Pseudoligand Interaction in the C222$_1$ PHD-HD1 Complex

Molecular surface representation of hPHD-HD1, showing the interaction with the N-terminal PHD tail (in grey, cylinder mode) from the other complex of the asymmetric unit. N-terminal residues of hPHD fragment used in this complex are indicated.

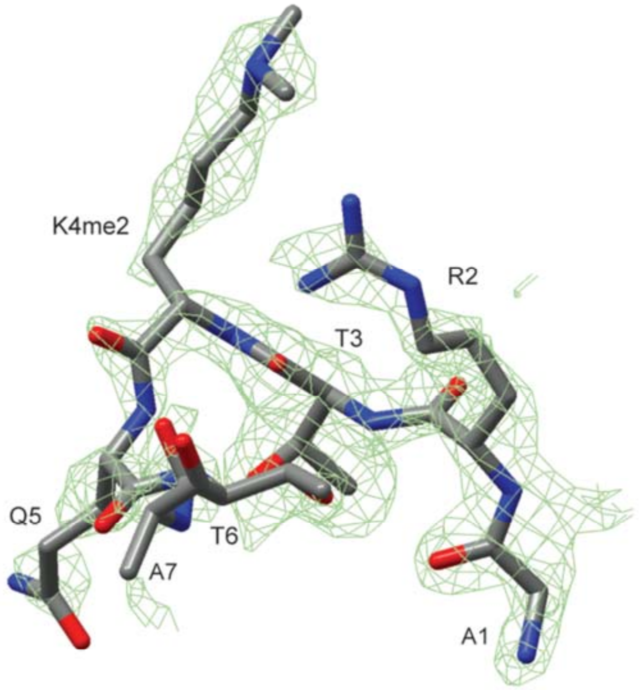

suppl. Fig.4 Fiedler et al.

Figure S4. Structural Model of the 9-mer H3K4me2 Peptide

Cylinder representation of the structure of the H3K4me2 tail in Tern1. A section of the unbiased difference map after molecular replacement is shown. All structured residues of the histone H3 tail (A1-A7) are labeled. Note that the structure of the peptide was the same in Tern2 (containing an 18-mer H3R2me2aK4me2 peptide).

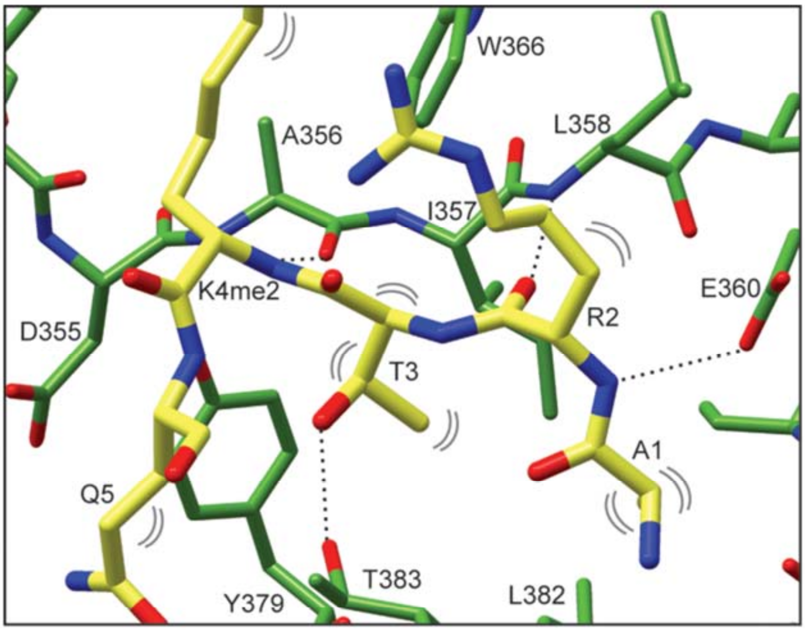

suppl. Fig.5 Fiedler et al.

Figure S5. Details of Structural Interactions between the Two Cavities

Residues A1 to Q5 of H3K4me2 (from Tern1) are shown in yellow, and interacting PHD residues (labeled) in green cylinder style; dotted lines, H-bonds; double brackets, hydrophobic interactions.

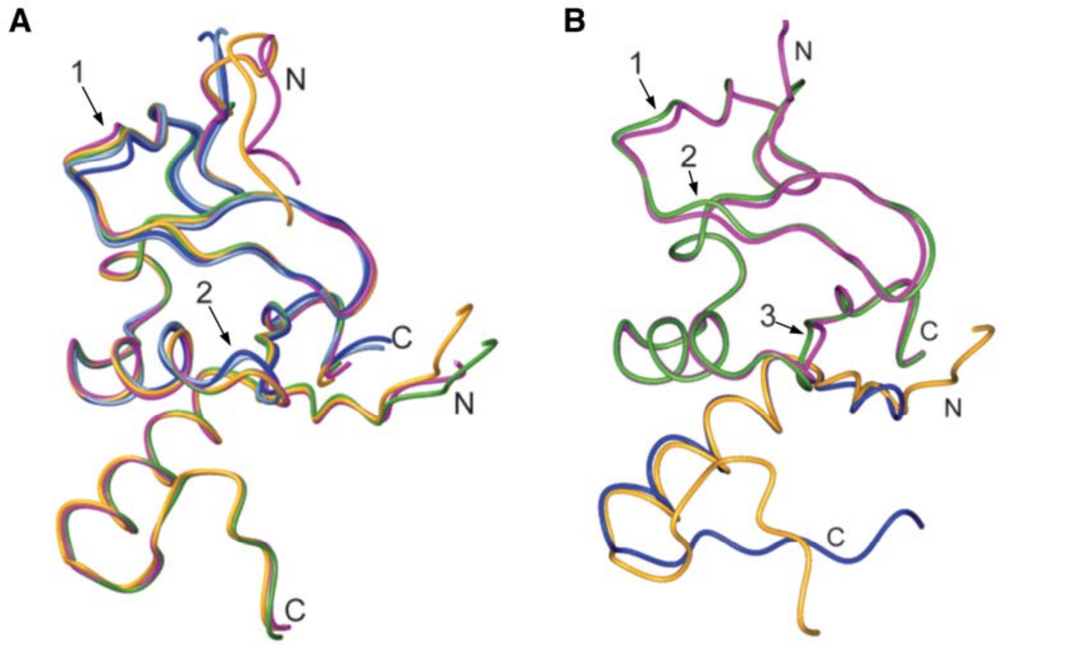

suppl. Fig.6 Fiedler et al.

Figure S6. Superimpositions of hPHD-HD1 Complex with Free hPHD Finger and with Ternary Complex

Backbones of free hPHD (Nakamura et al., 2007) and of various hPHD-HD1 and H3K4me2-hPHD-HD1 complexes were superimposed, revealing local conformational changes (indicated by arrows). (A) hPHD_W366F-HD1, magenta; hPHD-HD1 WT1, green; hPHD-HD1 WT2 (space group C222$_1$), orange; free PHD molecule A, dark blue; free PHD molecule B, light blue. (B) hPHD-HD1 WT1 (hPHD, green; HD1, orange), ternary complex Tern1 (hPHD, magenta; HD1, blue).
